# Antibiofilm-coated intramedullary nailing for tibial and femoral diaphyseal fractures at high-risk infection: outcomes of 103 consecutive patients

**DOI:** 10.1186/s10195-026-00967-x

**Published:** 2026-09-16

**Authors:** Giovanni Vicenti, Filippo Migliorini, Michele Loiodice, Alessandro Scarpino, Priscilla D’attis, Luise Schäfer, Giulia Colasuonno, Giuseppe Solarino

**Affiliations:** 1 Orthopaedic and Trauma Unit, Department of Translational Biomedicine and Neuroscience (DiBraiN), School of Medicine, University of Bari Aldo Moro, AOU Consorziale ‘Policlinico’, 70124 Bari, Italy; 2 Department of Trauma and Reconstructive Surgery, University Hospital Halle, Martin-Luther University Halle-Wittenberg, Ernst-Grube-Street 40, 06097 Halle (Saale), Germany; 3 Department of Life Sciences, Health, and Health Professions, Link Campus University, 00165 Rome, Italy; 4 Department of Orthopaedic and Trauma Surgery, Eifelklinik St. Brigida, Kammerbruchstr. 8, 52152 Simmerath, Germany; 5 Orthopaedic and Trauma Unit, ‘Monsignore Raffaele Dimiccoli’, 70051 Barletta, Italy

**Keywords:** Intramedullary nailing, Fracture-related infection, Bactiguard, Antibacterial coating, Tibial fracture, Femoral fracture, Biofilm, High-risk fracture, FRI prevention

## Abstract

**Background:**

Fracture-related infection (FRI) is a devastating complication of intramedullary nailing of tibial and femoral diaphyseal fractures. The present study evaluates the clinical efficacy of an antibacterially coated intramedullary nail in patients with high-risk tibial and femoral diaphyseal fractures. Open fractures, fractures requiring fasciotomy or closed fractures with Tscherne grade II or III soft-tissue damage were considered fractures at high risk of infection.

**Methods:**

A single-centre observational study was conducted between January 2022 and December 2024, in accordance with the Strengthening the Reporting of Observational Studies in Epidemiology (STROBE) guidelines. Patients who underwent definitive intramedullary nailing with Bactiguard (Bactiguard AB, Tullinge, Sweden)-coated Natural Nail (Zimmer Biomet, Warsaw, IN, USA) for tibial or femoral diaphyseal fractures at high risk of infection were included. The primary outcome was the incidence of FRI, defined according to the internationally validated consensus criteria. Secondary outcomes included time to union, rates of nonunion, malunion, unplanned secondary procedures and functional outcomes at a minimum follow-up of 12 months.

**Results:**

A total of 103 patients were enrolled: 55 with tibial fractures and 48 with femoral fractures. The combined cohort comprised 90 males and 13 females, with a median age of 40 years (tibial) and 32.5 years (femoral). Open fractures accounted for 38.2% and 16.7% of tibial and femoral cases, respectively; the remaining patients presented closed fractures with severe soft-tissue involvement (Tscherne grade II–III). FRI was diagnosed in 6 of 103 patients (5.8%): 4 of 55 in the tibial cohort (7.3%) and 2 of 48 in the femoral cohort (4.2%). All FRI cases were successfully managed without sepsis or limb amputation. Fracture union was achieved in 94.5% of tibial and 95.8% of femoral cases, with mean times to union of 9.8 ± 3.2 and 8.4 ± 2.3 months, respectively. No malunion was recorded in either cohort. Open reduction was significantly associated with FRI in both the tibial (*p* = 0.001) and femoral (*p* = 0.03) cohorts; articular extension of the fracture was associated with FRI in femoral fractures (*p* = 0.01).

**Conclusions:**

Intramedullary nailing with a Bactiguard-coated implant yielded an overall FRI rate of 5.8% in a consecutive series of 103 high-risk tibial and femoral diaphyseal fractures. Open reduction was identified as a significant independent risk factor for FRI in both cohorts. These findings are hypothesis-generating and support the rationale for prospective, controlled studies to establish the efficacy of biofilm-inhibiting coatings in orthopaedic trauma.

## Introduction

Tibia and femur diaphyseal fractures are common injuries usually caused by high-energy trauma [1–3]. If not promptly recognised and appropriately managed, these injuries may result in life-threatening complications, particularly in patients with polytrauma, owing to severe haemorrhage, associated visceral injuries and systemic complications [4–7]. Soft-tissue conditions are a critical determinant in the management of long-bone fractures [8, 9]. Historically, soft-tissue involvement has been classified according to the Gustilo–Anderson system for open fractures and the Tscherne classification for closed fractures [10, 11]. These classifications help guide early emergency decision-making towards damage-control strategies. The current standard for the definitive management of diaphyseal fractures of the femur and tibia remains intramedullary nailing [12–15]. Nonunion and fracture-related infection (FRI) are the two main complications of the intramedullary nail. In femoral fractures, nonunion rates range from 1.7% to 7.5% [16–18]. The incidence of FRI in femoral fractures increases from approximately 2% in closed injuries to 10–17% in open fractures or following high-energy trauma [19, 20]. FRI after intramedullary nailing remains a concern [21]. The reported incidence of FRI and nonunion in tibial fractures is approximately 2% and 12%, respectively [21, 22].

FRI is associated with considerable morbidity, potentially leading to sepsis, limb amputation and death [23–25]. Patients who have sustained high-energy trauma or present significant soft-tissue compromise are more prone to develop FRI [26]. One of the major factors complicating the eradication of FRI is the formation of bacterial biofilm on the implant surface [27, 28]. In this context, implementing strategies to mitigate the risk of implant-related infection is necessary.

The present study investigates the clinical efficacy of a coated intramedullary nail in high-risk scenarios involving femoral and tibial diaphyseal fractures. The purpose of the present study was to evaluate the time to union and the incidence of FRI and fracture nonunion following intramedullary nailing with a Bactiguard-coated implant in a consecutive series of high-risk tibial and femoral diaphyseal fractures.

## Methods

The present study was conducted in accordance with the Strengthening the Reporting of Observational Studies in Epidemiology (STROBE) [29]. This study was conducted at the Department of Orthopaedic and Trauma Surgery of the University Hospital of Bari, Italy, between January 2022 and December 2024. The present study was approved and registered by the Ethics Committee of the University Hospital of Bari, Italy (712, 22/05/2023) and conducted in accordance with the principles of the Declaration of Helsinki and its subsequent amendments. All patients understood the nature of their treatment and provided written consent to use their clinical and imaging data for research purposes.

### Study protocol

**Fig. 1:**
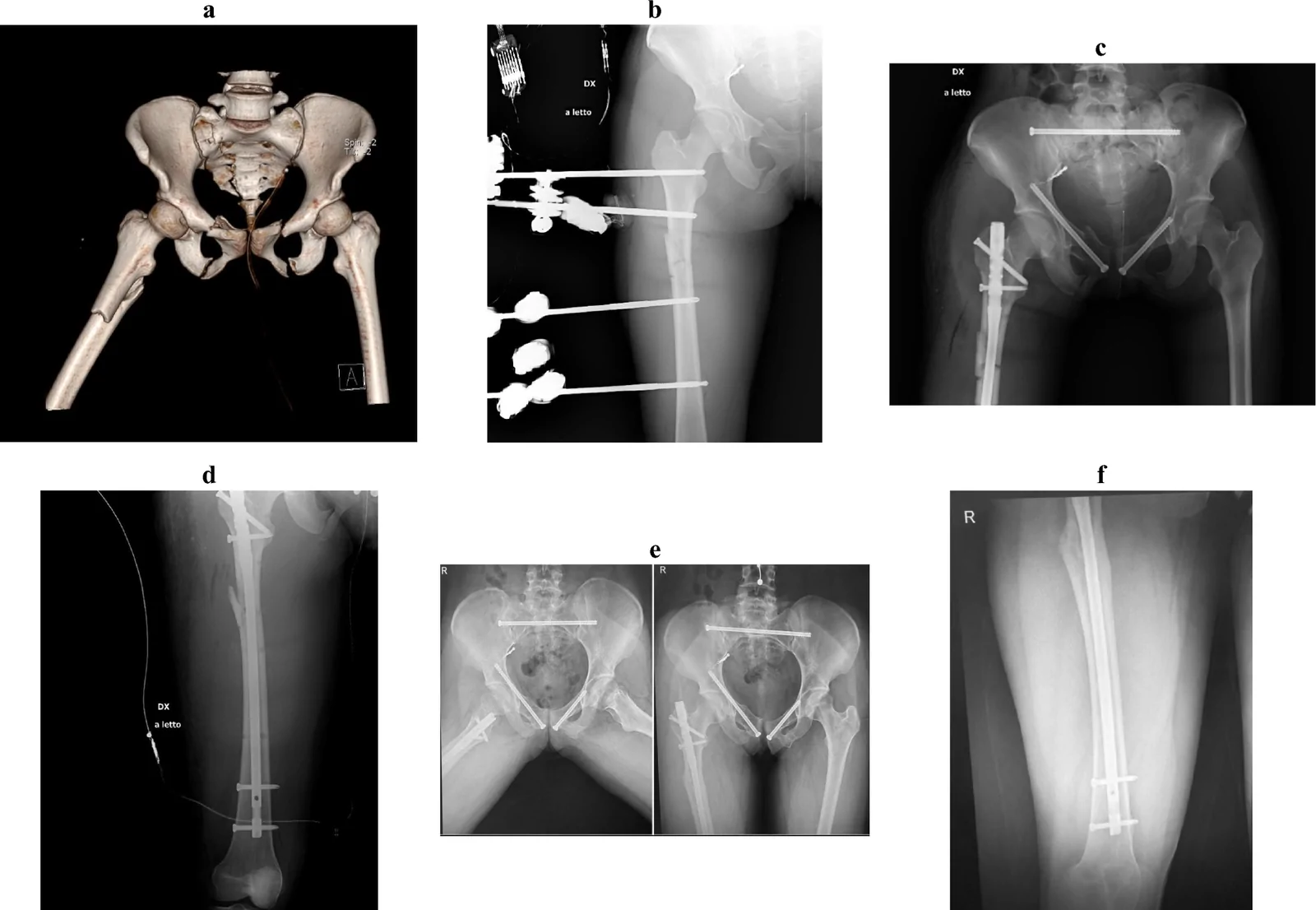
Representative case of a 19-year-old woman with a high-energy AO/OTA 32-B2 femoral shaft fracture associated with an unstable pelvic ring injury, managed with a Bactiguard-coated intramedullary nail. (**a**) Preoperative three-dimensional computed tomography reconstruction demonstrating the femoral and pelvic injuries. (**b**) Temporary damage-control external fixation of the femur. (**c**, **d**) Immediate postoperative anteroposterior and lateral radiographs after definitive fixation with a Bactiguard-coated intramedullary nail and concomitant pelvic stabilisation. (**e**, **f**) Radiographs obtained at the 18-month follow-up, demonstrating fracture union and maintained alignment without implant-related complications

**Fig. 2:**
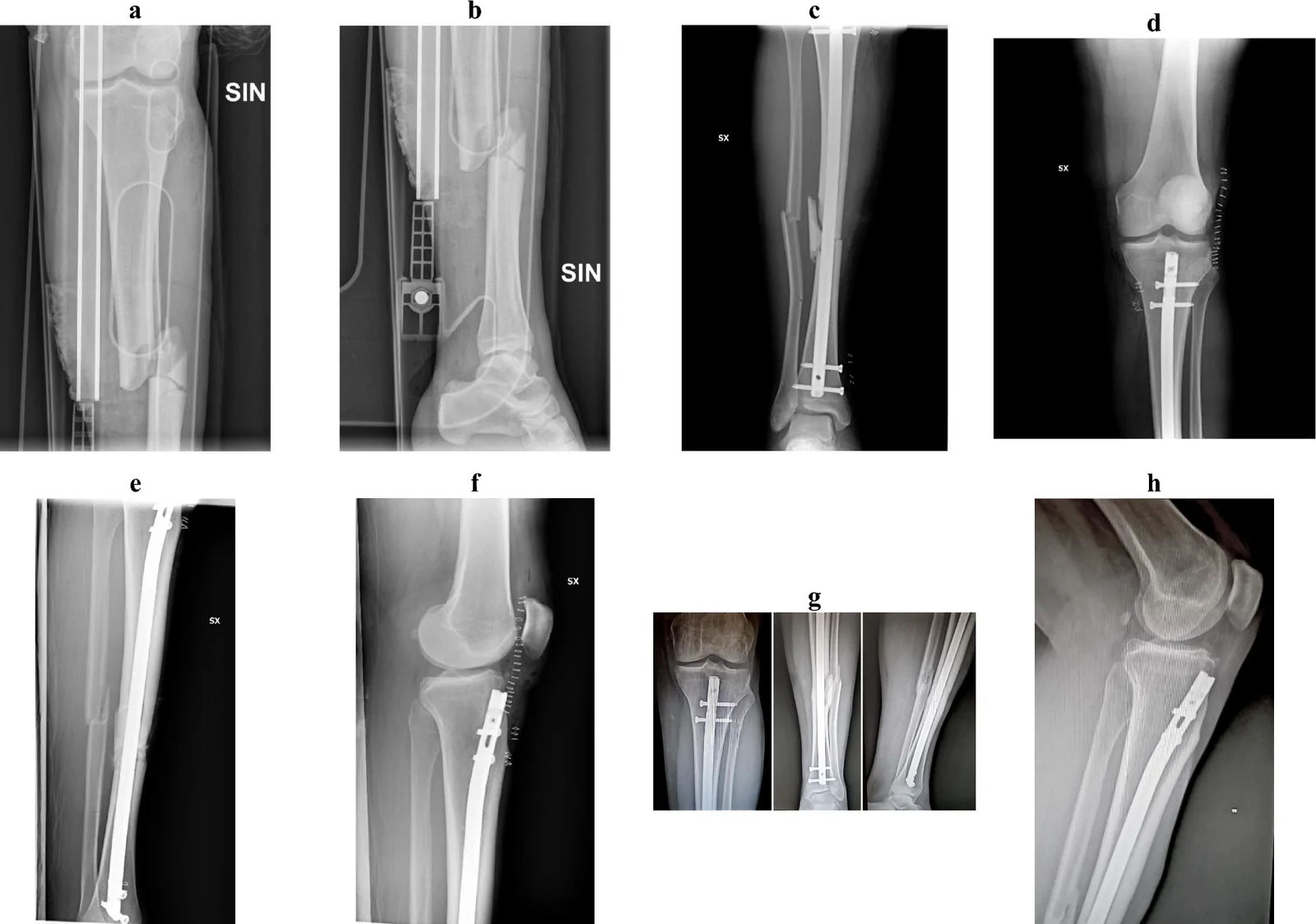
Representative case of a 27-year-old man with an AO/OTA 42-A2 tibial shaft fracture treated with a Bactiguard-coated intramedullary nail. (**a**, **b**) Preoperative anteroposterior and lateral radiographs. (**c**–**f**) Immediate postoperative radiographs following definitive fixation with a Bactiguard-coated intramedullary nail. (**g**, **h**) Radiographs obtained at the 20-month follow-up, demonstrating complete fracture union, restoration of alignment and stable implant fixation

All patients who underwent intramedullary nailing with Bactiguard (Bactiguard AB, Tullinge, Sweden)-coated Natural Nail (Zimmer Biomet, Warsaw, IN, USA) for femoral (Fig. 1) and tibial (Fig. 2) diaphyseal fractures at high risk of complications were included in the present study. Eligibility criteria were defined a priori for both the tibial and femoral cohorts. Patients who sustained a diaphyseal fracture of the tibia or femur were treated with intramedullary nailing as a definitive fixation method. All patients underwent clinical and radiological follow-up for at least 12 months. The study population was restricted to patients considered at high risk for infection, including those presenting with open fractures, those requiring fasciotomy and those with closed fractures associated with significant soft-tissue injury classified as Tscherne grade II or III.

### Data collection

Fractures were classified according to the AO/OTA classification system. The Injury Severity System [30] was used to assess the patient at admission. The Gustilo–Anderson classification was used to grade the severity of open fractures and the Tscherne classification was used to grade soft-tissue damage in closed fractures. Data on patient demographics, medical history, injury mechanisms, associated injuries and fracture classification were collected and analysed. Furthermore, information on the time from injury to surgery, from injury to external fixation or skeletal traction, from surgery to clinical and radiological bony union, intensive care unit (ICU) admission and the occurrence of local or systemic complications was collected. For each patient, the time to union, the rate of nonunion and FRI were recorded.

### Patient management

Open fractures were managed by the same team in accordance with the British Orthopaedic Association Standards for Trauma (BOAST) 4 guidelines [31], including prompt empiric antibiotic administration, thorough surgical debridement and early soft-tissue coverage. For closed fractures classified as Tscherne type II, trans-calcaneal traction was used for tibial fractures and trans-tibial traction for femoral fractures. Closed fractures classified as Tscherne type III were managed using an external fixator as damage control. Surgical bony fixation was performed by the same team. All closed fractures received perioperative antibiotic prophylaxis at the induction of anaesthesia, consisting of a single intravenous dose of 2 g cefazolin. Patients were positioned supine on a radiolucent operating table with the affected extremity draped free. Intramedullary nailing was performed with the knee in a semi-extended position via a suprapatellar approach or with the knee flexed using a sterile, draped triangular support for an infrapatellar approach. For the femur, a standard lateral approach to the great trochanter was performed. Canal reaming was performed to 1.5 mm beyond the selected nail diameter. All implants were statically locked both proximally and distally. Thromboprophylaxis with low-molecular-weight heparin was administered for 4 weeks [32]. Immediate postoperative unrestricted range of motion was allowed and weight-bearing as tolerated was encouraged, except in patients with concomitant injuries that precluded early mobilisation.

### Follow-up

After hospital discharge, all patients were followed for a minimum of 12 months. Fracture union was defined as painless full weight-bearing, combined with radiographic evidence of healing, as indicated by a Modified Radiographic Union Score for Tibial fractures (mRUST) greater than nine [33]. The mRUST is a validated radiographic scoring system that evaluates fracture healing by assessing callous formation and cortical bridging on the four cortices visible on anteroposterior and lateral radiographs, with each cortex graded from 1–4, resulting in a total score ranging from 4–16 [34, 35]. Malunion was defined as the presence of an angular or rotational deformity of ≥ 10° and/or limb shortening of ≥ 2 cm. FRIs were diagnosed according to previously published and validated diagnostic criteria [23, 36]. Fracture healing was considered uncomplicated when union was achieved within 6 months in the absence of local complications and without the need for secondary surgical procedures.

### Statistical analysis

The statistical analyses were performed using IBM SPSS Statistics version 25. Continuous variables were summarised as means with standard deviations and as medians with ranges. Categorical variables were reported as absolute values with corresponding percentages. Comparisons between groups were performed using the chi-square test or Fisher’s exact test, as appropriate. Statistical significance was defined as a *p*-value < 0.05.

## Results

### Patient demographics

A total of 103 patients who underwent intramedullary nailing with a Bactiguard-coated Natural Nail for tibial (*n* = 55) or femoral (*n* = 48) diaphyseal fractures at high risk of complications were enrolled between January 2022 and December 2024. The combined cohort comprised of 90 male and 13 female patients. The median age was 40 years (range 17–89) in the tibial cohort and 32.5 years (range 16–90) in the femoral cohort. Road traffic accidents were the predominant mechanism of injury in both groups, accounting for 61.8% and 87.5% of cases, respectively, with motorcycle crashes representing the single most frequent subgroup overall. Patient demographics, injury mechanisms and comorbidities are summarised in Table 1.

**Table 1:** Patient demographics, mechanism of injury and comorbidities for both cohorts

Variable	Tibia (*n* = 55)	Femur (*n* = 48)
Demographics		
Male/Female	50/5	40/8
Age – median, range (years)	40 (17–89)	32.5 (16–90)
Age – mean ± standard deviation (SD) (years)	41.2 ± 16.8	37.5
Mechanism of injury		
Fall	18 (32.7%)	6 (12.5%)
Simple fall	13 (23.6%)	6 (12.5%)
Fall from height	5 (9.1%)	0 (0%)
Road traffic accident	34 (61.8%)	42 (87.5%)
Motorcycle	17 (30.9%)	21 (43.8%)
Car/truck	7 (12.7%)	12 (25.0%)
Pedestrian	8 (14.5%)	6 (12.5%)
Bicycle	2 (3.6%)	3 (6.3%)
Struck by/against	3 (5.5%)	0 (0%)
Comorbidities and risk factors		
Severe trauma (injury severity score [ISS] > 15)	8 (14.5%)	10 (20.8%)
Smoking	22 (40.0%)	14 (29.2%)
Diabetes mellitus	11 (20.0%)	8 (16.7%)
High BMI	8 (14.5%)	9 (18.8%)
Alcohol use	3 (5.5%)	2 (4.2%)
Drug use	2 (3.6%)	2 (4.2%)

### Tibial intramedullary nailing

Fracture union was achieved in 52 of 55 patients (94.5%), with a mean time to union of 9.8 ± 3.2 months. The median mRUST score at the last follow-up was 11 (range, 6–12). Uncomplicated union was observed in 40 patients (72.7%). Three patients (5.5%) developed non-union. No malunion was recorded.

FRI was diagnosed in 4 of 55 patients (7.3%). Among the 21 open fractures, 3 (14.3%) developed FRI, all of which were Gustilo–Anderson grade IIIA; notably, none of the four grade IIIB fractures developed FRI. Among the 32 patients who underwent damage-control external fixation, 3 developed FRI; the mean time to conversion was 20 days in patients who developed FRI versus 17 days in those who did not – a difference that did not reach statistical significance, likely owing to the limited sample size. Chi-square analysis identified a statistically significant association between open reduction and FRI occurrence (*p* = 0.001). Full fracture characteristics, surgical details and outcomes are presented in Table 2.

**Table 2:** Fracture characteristics, surgical management and clinical outcomes of the tibial cohort

Variable	Tibia (*n* = 55)
Fracture characteristics	
Laterality – right/left	27 (49%)/28 (51%)
AO type A/B/C	21 (38.2%)/24 (43.6%)/10 (18.2%)
Open fractures	21 (38.2%)
Gustilo–Anderson type I	5 (23.8%)
Gustilo–Anderson type II	6 (28.6%)
Gustilo–Anderson type IIIA	6 (28.6%)
Gustilo–Anderson type IIIB	4 (19.0%)
Tscherne grade II/III (closed fractures)	18 (52.9%)/16 (47.1%)
Articular extension	11 (20.0%)
Bone loss	6 (10.9%)
Surgical management	
External fixation (damage control)	32 (58.2%)
Time to conversion – median, range (days)	15 (5–41)
Time to conversion – mean ± SD (days)	17.8 ± 9.3
Skeletal traction	10 (18.2%)
Time to conversion – mean ± SD (days)	4.1 ± 1.4
Emergency fasciotomy	2 (3.6%)
Time to primary skin closure – mean ± SD (days)	17.5 ± 2.5
Time to definitive fixation – median, range (days)	10 (2–41)
Mean ± SD (days)	13.0 ± 9.9
Wound coverage required	21 (38.2%)
Primary closure	17 (30.9%)
Gastrocnemius flap	2 (3.6%)
Free flap	2 (3.6%)
Infrapatellar approach	43 (78.2%)
Suprapatellar approach	12 (21.8%)
Closed reduction	42 (76.4%)
Open reduction	12 (21.8%)
Frame-assisted reduction	3 (5.5%)
Poller screws	2 (3.6%)
Additional procedures (local antibiotics, fibula fixation, Masquelet, metaphyseal screws)	16 (29.1%)
Outcomes	
Hospital stay – median, range (days)	15 (5–60)
Mean ± SD (days)	21.9 ± 20.3
Follow-up – median, range (months)	12 (12–40)
Union rate	52/55 (94.5%)
Time to union – median, range (months)	8.5 (5–14)
Mean ± SD (months)	9.8 ± 3.2
mRUST score – median, range	11 (6–12)
Mean ± SD	10.0 ± 1.5
Uncomplicated union	40 (72.7%)
Nail dynamisation	11 (20.0%)
Unplanned secondary procedures	11 (20.0%)
Non-union	3 (5.5%)
Malunion	0 (0%)
VTE	1 (1.8%)
Fracture-related infection (FRI)	4 (7.3%)
Reduced knee ROM at last follow-up	11 (20.0%)
Knee pain/discomfort at last follow-up	12 (21.8%)
Reduced ankle ROM at last follow-up	12 (21.8%)
Ankle pain/discomfort at last follow-up	7 (12.7%)

FRI fracture-related infection, mRUST modified Radiographic Union Score for Tibial fractures, ROM range of motion, VTE venous thromboembolism

### Femoral intramedullary nailing

Fracture union was achieved in 46 of 48 patients (95.8%), with a mean time to union of 8.4 ± 2.3 months. The median mRUST score was 11 (range 6–12). Uncomplicated union was achieved in 41 patients (85.4%). Two patients (4.2%) developed non-union. No malunion occurred. FRI was diagnosed in 2 of 48 patients (4.2%). Notably, none of the eight patients with open femoral fractures developed FRI. Both femoral FRI occurred in patients with closed fractures, suggesting the potential influence of patient-specific risk factors independent of the injury pattern. Chi-square analysis identified a statistically significant association between open reduction and FRI incidence (*p* = 0.03), as well as between articular extension of the fracture and FRI occurrence (*p* = 0.01). Full fracture characteristics, surgical details and outcomes are presented in Table 3.

**Table 3:** Fracture characteristics, surgical management and clinical outcomes of the femoral cohort

Variable	Femur (*n* = 48)
Fracture characteristics	
Laterality – right/left	23 (48%)/25 (52%)
AO type A/B/C	19 (39.6%)/10 (20.8%)/19 (39.6%)
Open fractures	8 (16.7%)
Gustilo–Anderson type I	1 (12.5%)
Gustilo–Anderson type II	5 (62.5%)
Gustilo–Anderson type IIIA	2 (25.0%)
Gustilo–Anderson type IIIB	0 (0%)
Tscherne grade II/III (closed fractures)	17 (42.5%)/23 (57.5%)
Articular extension	12 (25.0%)
Bone loss	2 (4.2%)
Surgical management	
External fixation (damage control)	37 (77.1%)
Time to conversion – median, range (days)	13 (3–40)
Time to conversion – mean ± SD (days)	15.2 ± 7.7
Skeletal traction	11 (22.9%)
Time to conversion – mean ± SD (days)	9.2 ± 7.0
ICU admission prior to definitive fixation	9 (18.8%)
Emergency fasciotomy	0 (0%)
Time to definitive fixation – median, range (days)	12.5 (3–45)
Mean ± SD (days)	14.9 ± 9.4
Wound coverage required	8 (16.7%)
Primary closure	8 (16.7%)
Anterograde nailing	22 (45.8%)
Retrograde nailing	26 (54.2%)
Closed reduction	32 (66.6%)
Open reduction	15 (31.3%)
Frame-assisted reduction	2 (4.2%)
Poller screws	2 (4.2%)
Additional procedures (local antibiotics, Masquelet, metaphyseal screws)	8 (16.7%)
Outcomes	
Hospital stay – median, range (days)	22 (7–45)
Mean ± SD (days)	21.1 ± 8.4
Follow-up – median, range (months)	12 (12–38)
Union rate	46/48 (95.8%)
Time to union – median, range (months)	9 (5–15)
Mean ± SD (months)	8.4 ± 2.3
mRUST score – median, range	11 (6–12)
Mean ± SD	10.0 ± 1.5
Uncomplicated union	41 (85.4%)
Nail dynamisation	6 (12.5%)
Unplanned secondary procedures	6 (12.5%)
Non-union	2 (4.2%)
Malunion	0 (0%)
VTE	0 (0%)
Fracture-related infection (FRI)	2 (4.2%)
Reduced knee ROM at last follow-up	6 (12.5%)
Knee pain/discomfort at last follow-up	9 (18.8%)
Reduced ankle ROM at last follow-up	2 (4.2%)
Ankle pain/discomfort at last follow-up	5 (10.4%)

FRI fracture-related infection, mRUST modified Radiographic Union Score (originally developed for tibial fractures), ROM range of motion, VTE venous thromboembolism

### Overall infection rate

Across both cohorts, FRI was diagnosed in 6 of 103 patients (5.8%): 4 of 55 in the tibial cohort (7.3%) and 2 of 48 in the femoral cohort (4.2%). All FRI cases were successfully managed without sepsis, limb amputation or infection-related mortality.

## Discussion

According to the main findings of the present investigation, intramedullary nailing with a Bactiguard-coated implant yielded an overall FRI rate of 5.8% in a consecutive series of 103 high-risk tibial and femoral diaphyseal fractures. High union rates and an absence of malunion further support the safety of this approach. Open reduction was identified as a significant independent risk factor for FRI in both cohorts. These findings are hypothesis-generating and support the rationale for prospective, controlled studies to establish the efficacy of biofilm-inhibiting coatings in orthopaedic trauma.

FRI is a major complication following intramedullary nailing of tibial and femoral fractures, especially in patients with low performance status [26, 37]. The management of FRI remains challenging, with unpredictable results [38–40]. Antibiotic-coated intramedullary nails have emerged as a promising option to reduce the rate of FRIs following tibia and femur fractures [41–43]. These devices seem to significantly reduce infection rates, hospital lengths of stay and reoperation rates in open fractures [41, 44]. However, concerns persist about their widespread use, particularly regarding the potential for antimicrobial resistance [39, 45]. Considering both coated and uncoated nails, the overall incidence of FRI was 9.4%. The coated nails seemed to report a lower incidence [42, 43]. In the current literature, there are few data on the incidence of FRI after the 2020 consensus [23] and only cumulative data are available for tibia and femur fractures, with unclear criteria for FRI attribution. Several clinical studies investigated the incidence of surgical site infection in patients with tibia and femoral fractures, with a range from 7.6% to 11.8% [8, 46]. In 2019, Saleeb et al. [20] wrote a review on the incidence of deep infections, non-union and malunion in open diaphyseal femur fractures treated with intramedullary nails: the results showed a union rate of 97%, deep infection in 6% (mainly Gustilo III) and superficial infection in 5.6%, considering the 17 studies analysed. In the present study, 103 high-risk patients with diaphyseal fractures of the tibia (55) and femur (48) treated with intramedullary nails were considered. Considering the 55 patients with tibial fractures, the FRI rate was 7.3% (four patients). In particular, among patients with ISS > 15 (8 of 55), the incidence of FRI was low (only one patient). A total of 32 patients underwent damage control with an external fixator, of whom 3 developed FRI. Time to conversion is crucial: in patients who did not develop FRI, the average time was 17 days, whereas in the three patients who developed FRI, it was 20 days. However, this data is not statistically significant, likely given the small population size. Further investigations are needed to evaluate the association between the time to conversion and FRI occurrence. Only 3 patients developed FRI of the 21 patients in the open tibial fractures group. These three patients had a Gustilo grade IIIA exposure. In this population, four IIIB GA fractures occurred; none developed FRI, possibly because of the acute soft-tissue coverage provided during damage control with an external fixator. Additional analyses should investigate this point on a larger scale.

A lower incidence of FRI has been reported in femoral diaphyseal fractures, possibly attributable to the anatomical location of the bone and the more extensive coverage provided by the surrounding soft tissues. This data, along with previously published reports, might partially explain the lack of evidence on this matter [19, 47]. A total of 10 out of 48 patients with femoral fractures sustained severe trauma (ISS > 15). These patients underwent a damage-control strategy to optimise soft-tissue management. Temporary external fixation was performed in 37 patients: 8 with open fractures, 23 with closed Tscherne grade III fractures and 6 with closed Tscherne grade II fractures. The remaining 11 patients with closed Tscherne grade II fractures were managed with skeletal traction. None of the patients who had sustained an open fracture of the femur developed FRI. In contrast, FRI was observed only in patients with closed fractures, suggesting possible patient-specific factors rather than injury patterns. Closed reduction is preferred over open reduction to minimise the risk of FRI [48–50]. However, when the fracture extends to the joint, it requires anatomical open reduction, which is inherently associated with an increased risk of FRI [51]. For these reasons, we encourage mini-open or percutaneous reduction techniques when possible.

The present study has several limitations that should be considered when interpreting its findings. The absence of a concurrent control group represents the principal methodological constraint. All patients received Bactiguard-coated intramedullary nails, and no parallel cohort of patients receiving uncoated implants under the same surgical protocol and institutional setting was available for direct comparison. Consequently, causal inferences regarding the infection-preventive effect of the coating cannot be drawn and the reported FRI rates can only be contextualised against historical benchmarks derived from heterogeneous literature sources, a comparison that is inherently limited by differences in patient selection, fracture severity, surgical technique, antibiotic prophylaxis protocols and FRI diagnostic criteria across studies. The retrospective, single-centre design introduces an inherent risk of selection bias and limits the generalisability of the results to other institutional settings. The study was conducted at a single tertiary trauma centre by a dedicated surgical team, and the observed outcomes may reflect centre-specific expertise, patient volume and perioperative management protocols that may not be reproducible in lower-volume or non-specialist institutions. The relatively small sample size, particularly in subgroups defined by fracture severity, mechanism of injury and soft-tissue classification, limited the statistical power of subgroup analyses. Several comparisons of clinical relevance, such as the association between time to conversion from external fixation and FRI incidence, did not reach statistical significance likely owing to insufficient sample size rather than a true absence of effect. Larger, prospective studies will be required to validate these preliminary observations. Fourth, the minimum follow-up of 12 months, while appropriate for the primary outcome of fracture union and early infectious complications, may not be sufficient to capture late-onset FRI, implant-related sequelae or long-term functional outcomes. Late infections attributable to low-virulence microorganisms, which may manifest beyond the 12-month threshold, may have been missed in some patients. The Charlson Comorbidity Index was not systematically calculated for all patients, precluding a standardised, quantitative assessment of the overall comorbidity burden and its potential influence on infectious and healing outcomes. Although individual comorbidities were recorded and reported, the absence of a composite score limits the ability to adjust for baseline health status in comparative analyses. Microbiological data were not uniformly available across all FRI cases, preventing a comprehensive characterisation of causative pathogens and their resistance profiles. This precludes any assessment of the potential impact of the coating on the microbiological spectrum of infection or on the development of antimicrobial resistance, a concern raised in the context of coated implants and warranting dedicated investigation in future studies. Patient-reported outcome measures (PROMs) were not prospectively collected. The functional assessment was limited to clinician-reported parameters, namely knee and ankle range of motion and the presence of pain or discomfort at the last follow-up visit. The inclusion of validated PROMs, such as the EuroQol EQ-5D or the Lower Extremity Functional Scale, would have provided a more patient-centred evaluation of treatment outcomes and enhanced comparability with other published series.

## Conclusions

Intramedullary nailing with a Bactiguard-coated implant yielded an overall FRI rate of 5.8% in a consecutive series of 103 high-risk tibial and femoral diaphyseal fractures, comparing favourably with published benchmarks for conventional uncoated nailing in similar populations. High union rates and an absence of malunion further support the safety of this approach. Open reduction was identified as a significant independent risk factor for FRI in both cohorts. These findings are hypothesis-generating and support the rationale for prospective, controlled studies to establish the efficacy of biofilm-inhibiting coatings in orthopaedic trauma.

## Data Availability

The datasets generated and/or analysed during the current study are available from the corresponding author upon reasonable request.
